# Acute myocardial infarction with left main coronary artery ostial negative remodelling as the first manifestation of Takayasu arteritis: a case report

**DOI:** 10.1186/s12872-021-02376-w

**Published:** 2021-11-22

**Authors:** Shiqiang Zhou, Chao Gao, Fei Li

**Affiliations:** 1 Department of Cardiology, Xi’jing Hospital, Air Force Medical University, Changle West Road, Xi’an, 710032 China; 2 Department of Cardiology, Radboud University, Nijmegen, The Netherlands

**Keywords:** Coronary artery, Negative remodelling, Takayasu arteritis, Intravenous ultrasound, Case report

## Abstract

**Background:**

Takayasu arteritis is a chronic inflammatory disease involving the aorta and its major branches. Acute myocardial infarction rarely but not so much presents in patients with Takayasu arteritis, and the preferable revascularization strategy is still under debate.

**Case presentation:**

A 22-year-old female with Takayasu arteritis presented with acute myocardial infarction. Coronary angiography and intravenous ultrasound (IVUS) showed that the right coronary artery (RCA) was occluded and that there was severe negative remodelling at the ostium of the left main coronary artery (LMCA). The patient was treated by primary percutaneous transluminal coronary angioplasty (PTCA) with a scoring balloon in the LMCA, without stent implantation. After 3 months of immunosuppressive medication, the patient received RCA revascularization by stenting. There was progressive external elastic membrane (EEM) enlargement of the LMCA ostium demonstrated by IVUS at 3 and 15 months post-initial PTCA.

**Conclusion:**

Here, we report a case of Takayasu arteritis with involvement of the coronary artery ostium. Through PTCA and long-term immunosuppressive medication, we found that coronary negative remodelling might be reversible in patients with Takayasu arteritis.

## Background

Takayasu arteritis is a chronic nonspecific vasculitis with uncertain pathogenesis that mostly involves the aorta and its major branches and occasionally affects the coronary arteries. It has been reported that the pathological features of coronary artery involvement may be characterized by stenosis or occlusion, diffuse or focal coronary arteritis, and the formation of aneurysms [1]. Here, we describe a case of acute myocardial infarction due to Takayasu arteritis with coronary ostial negative remodelling.

## Case presentation

A 22-year-old female presented at our hospital with sudden-onset chest pain and was diagnosed with acute myocardial infarction. One month before admission, she attended our outpatient clinic with a chief complaint of exertional chest distress. The diagnosis of possible Takayasu arteritis was established, supported by systematic examination of computed tomography angiography, which showed annular thickening of the aortic root sinus and ascending aorta wall with mild stenosis; in addition, there was severe stenosis in the RCA and LMCA ostium. The patient had a EuroScore II of 1.29% and a Society of Thoracic Surgeons (STS) score of 1.06%. After discussion, the heart team recommended coronary artery bypass grafting, but the patient declined.

**Fig. 1:**
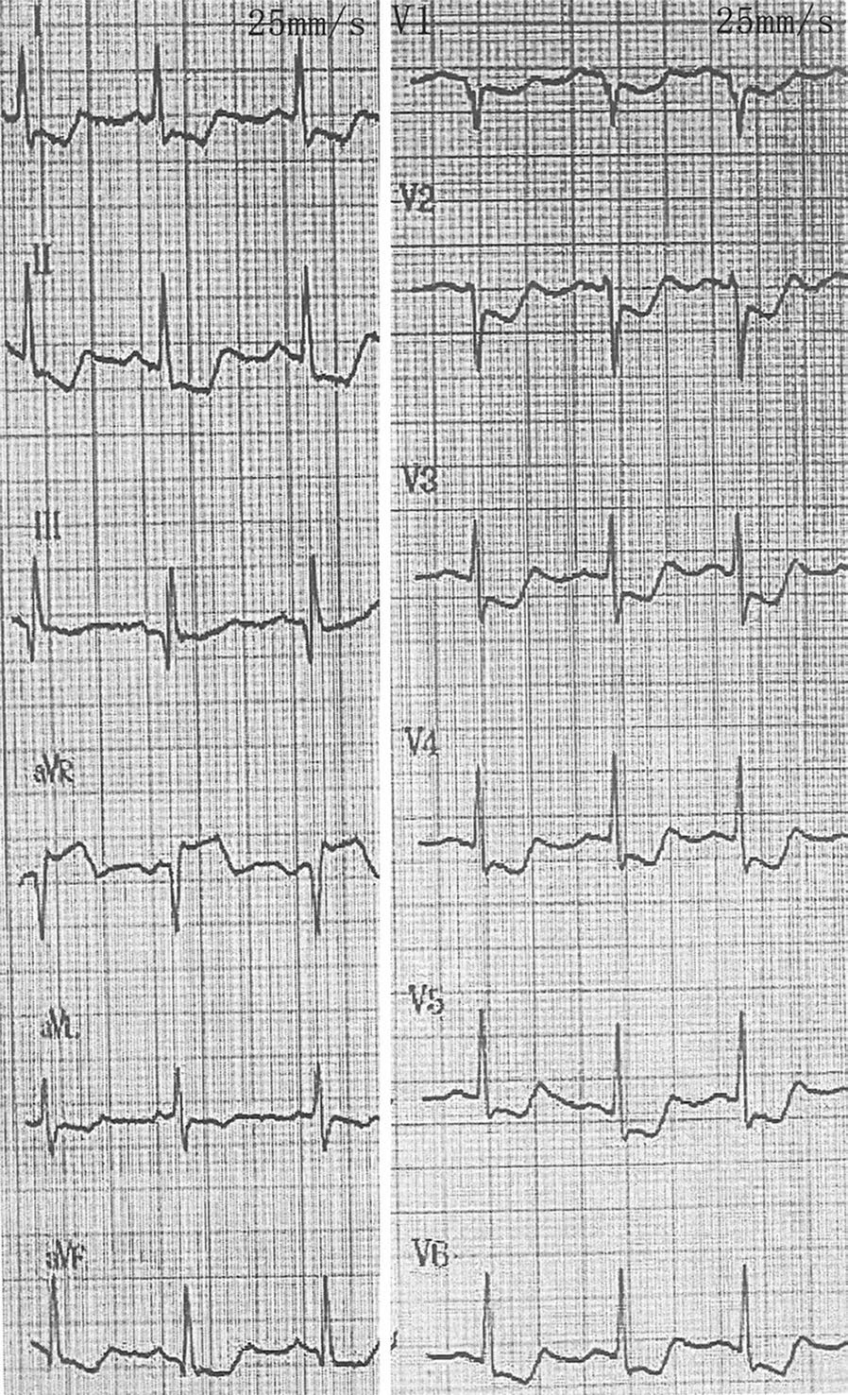
Electrocardiogram. The preoperative electrocardiogram showed ST-segment elevation in the aVR lead and ST-segment depression in the other leads

After admission for acute myocardial infarction, the patient experienced intermittent postinfarction angina. On physical examination, her blood pressure was 90/60 mmHg, with no significant difference between the arms. Peripheral arteries were equally palpable, and there were no vascular bruits or heart murmurs. Electrocardiogram revealed ST-segment elevation in the aVR lead and ST-segment depression in other leads (Fig. 1). Transthoracic echocardiography noted diffuse hypokinesia of the interventricular septum and anterior, lateral, posterior, and inferior walls of the left ventricle, with a left ventricular ejection fraction of 44%. Creatinine kinase isoenzyme, troponin I, and serum brain-type natriuretic peptide were 112 ng/ml (reference range, 0.3–4.0 ng/ml), 2.288 ng/ml (reference range, 0–0.03 ng/ml), and 1,189 pg/ml (reference range, < 125 pg/ml), respectively. There were no abnormal findings in laboratory tests for connective tissue disease. The high-sensitivity C-reactive protein level was 28.6 mg/L (reference range, 0–6 mg/L).

**Fig. 2:**
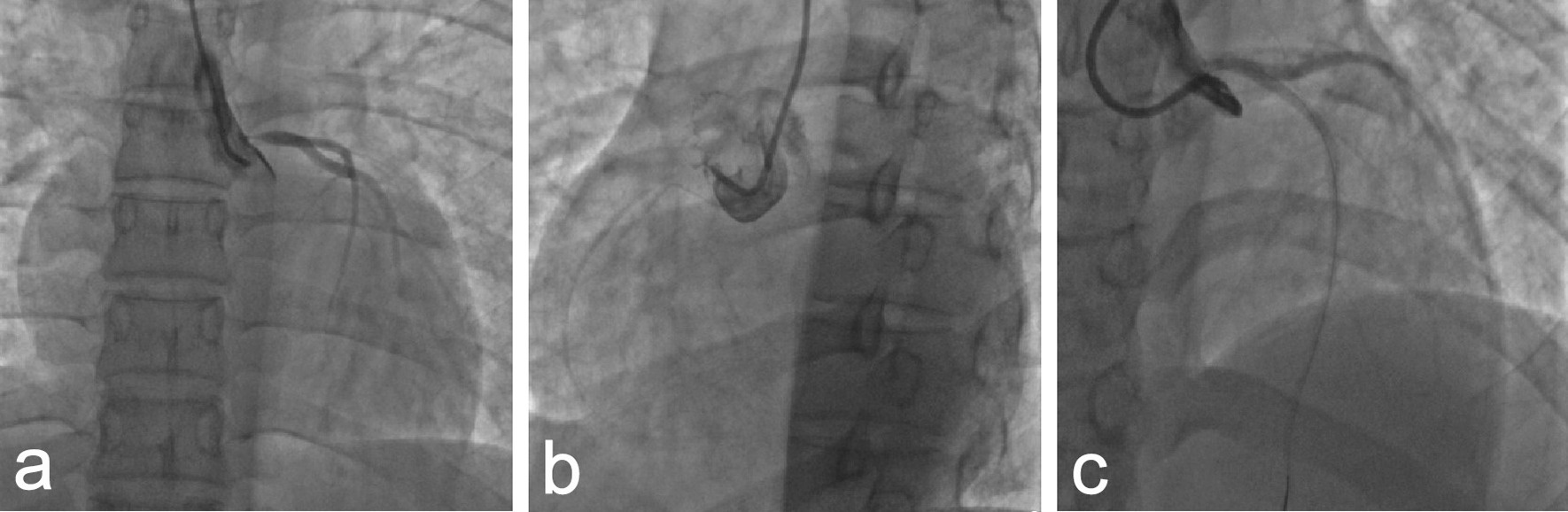
Coronary angiography. **a** Severe stenosis at the ostium of the left main coronary artery (LMCA). **b** The right coronary artery (RCA) was totally occluded, and the distal part of the vessel was visualized via collaterals. **c** The LMCA after angioplasty

**Fig. 3:**
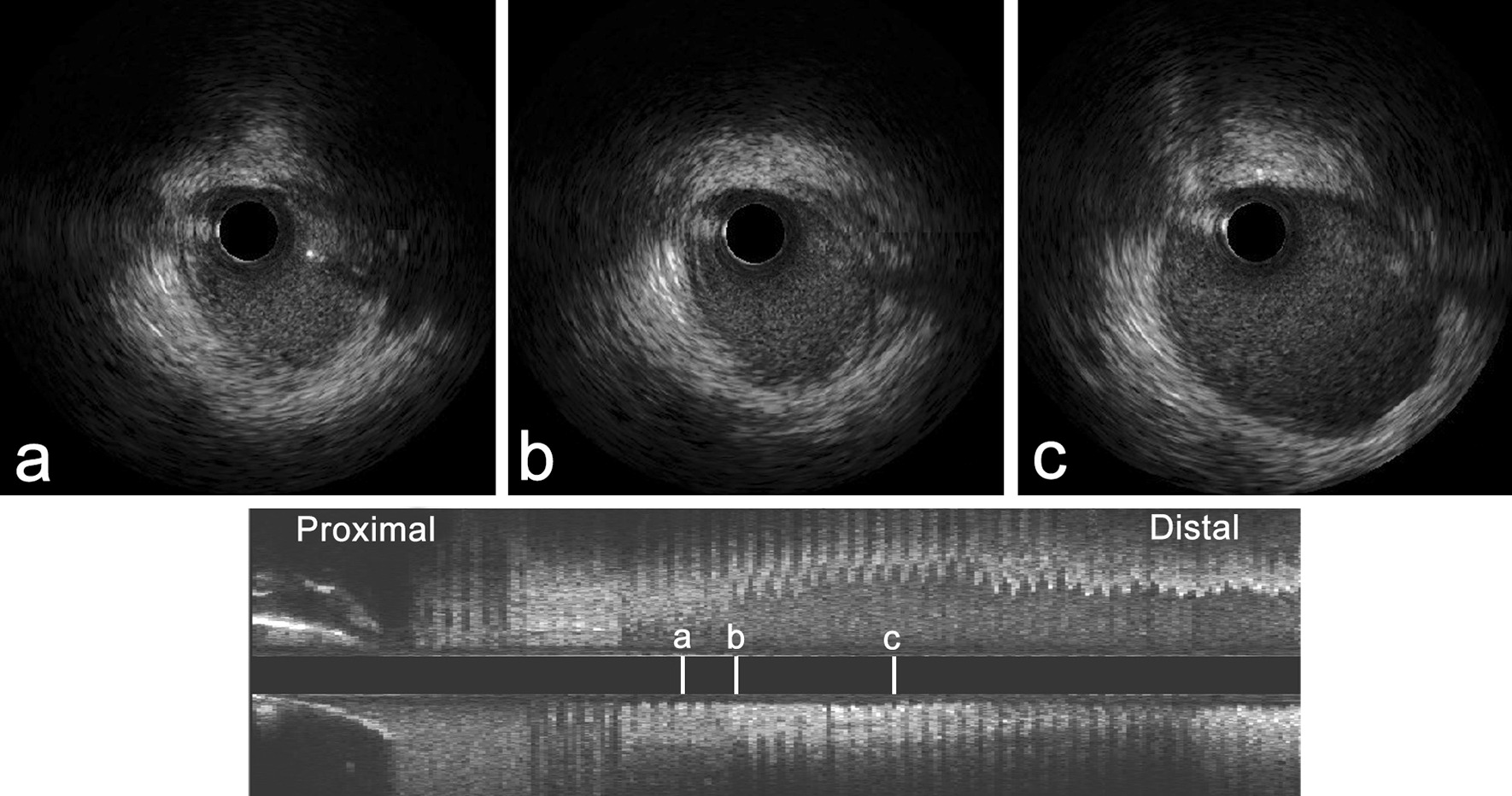
Intravenous ultrasound (IVUS) images. After the first percutaneous transluminal coronary angioplasty (PTCA), IVUS demonstrated that the external elastic membrane (EEM) areas of the ostium, proximal segment and middle segment of the LMCA were 7.11 (**a**), 8.34 (**b**) and 15.68 (**c**), respectively (in mm^2^). It also showed negative remodelling of the ostium of the LMCA (remodelling index = 0.45). The remodelling index was calculated by the ratio of the EEM areas of the middle LMCA segment to the LMCA ostium (7.11 mm^2^/15.68 mm^2^)

Nonselective coronary angiography showed severe stenosis at the ostium of the LMCA (Fig. 2a), and the RCA was totally occluded with collateral circulation (Fig. 2b). Angioplasty with a semicompliant balloon and scoring balloon was then performed to dilatate the stenosed LMCA ostium (Fig. 2c). Afterwards, intravascular imaging by IVUS showed that the minimal luminal area of the LMCA was 5.64 mm^2^, and the EEM area at the ostium of the LMCA was 7.11 mm^2^ (Fig. 3a). Meanwhile, there was negative remodelling of the ostium of the LMCA (Fig. 3a–c). The remodelling index was 0.45 (Fig. 3a, c).

**Fig. 4:**
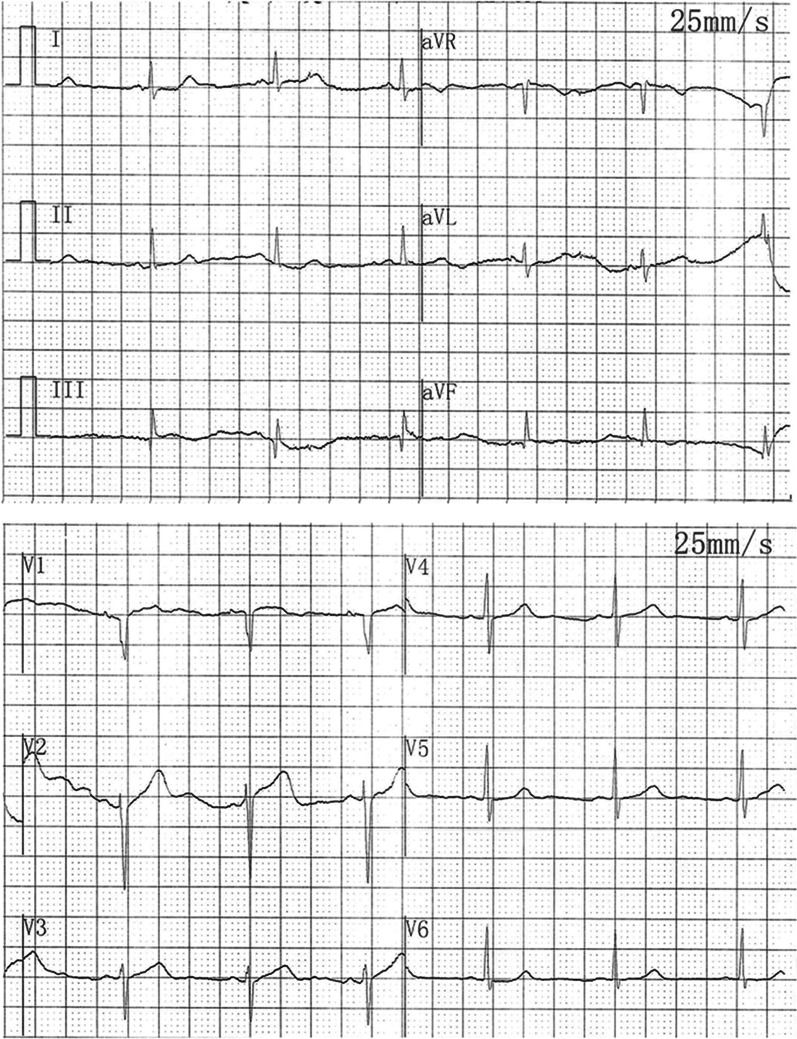
Electrocardiogram. The postoperative electrocardiogram showed no ST-segment depression

Postoperative electrocardiogram showed no ST-segment depression (Fig. 4). Aspirin, clopidogrel and low-molecular-weight heparin were given post-PTCA with close monitoring. The patient did not experience recurrence of angina and was then referred to the Department of Immunology for further diagnostic work-up and management. The test results of treponema pallidum antibody, early secreted antigen target 6-kDa protein and culture filtrate protein 10 were negative. Echocardiography showed a left ventricular ejection fraction of 53%. The diagnosis of active Takayasu arteritis was established, and immunosuppressive medications were prescribed. After a multidisciplinary specialist discussion, we decided to defer stent implantation into the LMCA and instead planned a staged percutaneous coronary intervention. The patient remained asymptomatic and was discharged with a prescription of prednisone 30 mg/day, methotrexate 10 mg/week, aspirin 100 mg/day, clopidogrel 75 mg/day and atorvastatin 20 mg/day.

**Fig. 5:**
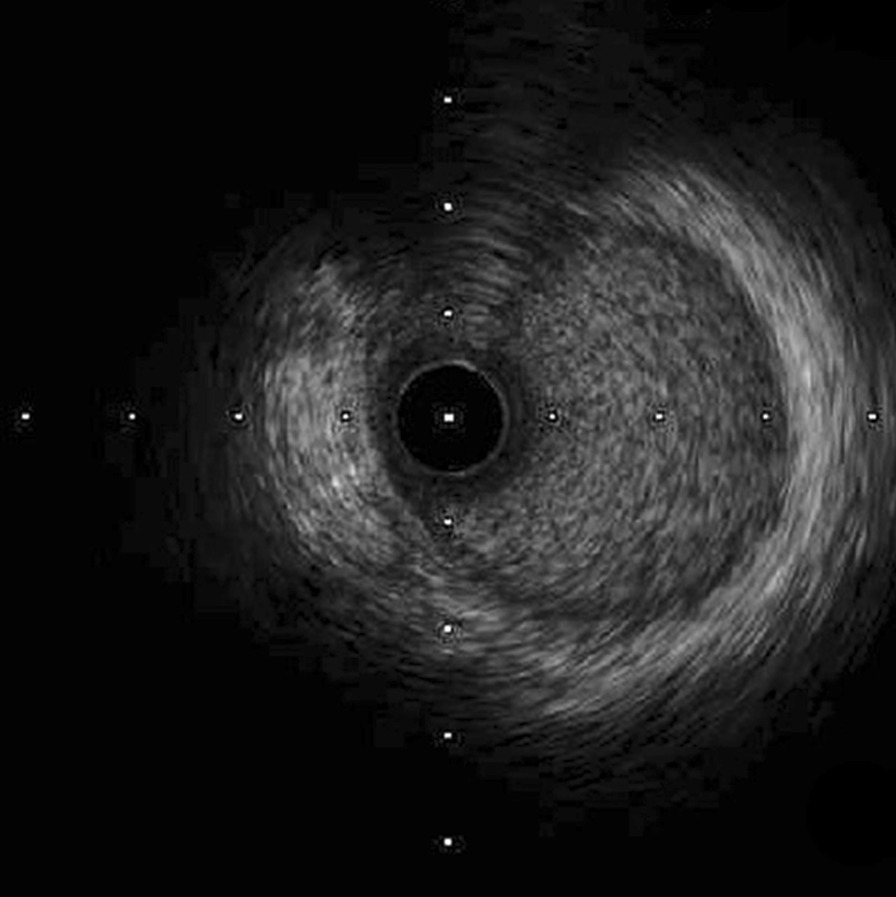
IVUS image. The result of follow-up IVUS examination at 3 months post-initial PTCA showed that the EEM area of the LMCA ostium was 11.74 (in mm^2^)

Three months later, the patient was rehospitalized for the staged percutaneous coronary intervention. IVUS showed that the minimal lumen area of LMCA was 5.92 mm^2^; the EEM area at the ostium of LMCA was 11.74 mm^2^ (Fig. 5). Although the minimal lumen area of LMCA did not reach 8.0 mm^2^, given that the EEM area enlarged compared with that at the initial PTCA, we decided to leave the LMCA unstented and revascularize the occluded RCA. To revascularize the RCA, we performed retrograde rewiring after a failed antegrade attempt. A sirolimus-eluting stent (Firehawk 3.0 × 13 mm, Shanghai MicroPort Medical Corporation, China) was implanted proximal to the RCA.

The patient was then scheduled for follow-up once every three months. She remained asymptomatic, and the results of laboratory examinations showed that the erythrocyte sedimentation rate and high-sensitivity C-reactive protein were in the normal range. There were no drug-related adverse events during the follow-up.

**Fig. 6:**
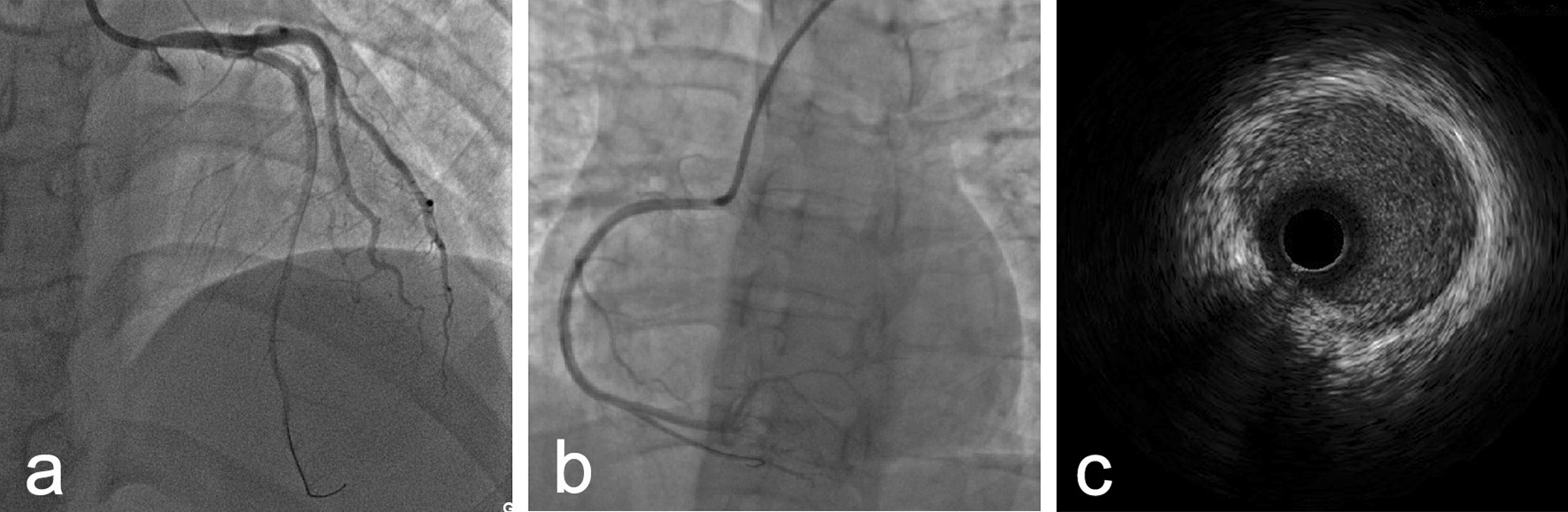
Coronary angiography and IVUS image. At 15 months follow-up after the first percutaneous coronary intervention, coronary angiography revealed regression of the LMCA ostium (**a**) and good patency of the RCA stent site (**b**), and IVUS showed that the EEM area at the ostium of LMCA was 12.85 (**c**) (in mm^2^)

At 15 months poststent implantation, we re-examined the patient with coronary angiography and IVUS. Coronary angiography showed regression of the ostium of the LMCA (Fig. 6a) and good patency of the site of the RCA stent (Fig. 6b). IVUS detected a progressive enlargement of the EEM area at the ostium of the LMCA. The minimal lumen area of the LMCA was 7.61 mm^2^ and the EEM area in the ostium of LMCA was 12.85 mm^2^ (Fig. 6c). There was minimal hyperplasia in the proximal RCA stent. Ultrasonography of the carotid artery, subclavian artery, and arteries of both lower limbs showed no positive findings.

## Discussion and conclusions

The incidence of coronary artery disease involving Takayasu arteritis is approximately 10–30% [2]. For most of these patients, coronary stenosis or occlusion is located at the coronary ostia and proximal segments of the coronary arteries [3, 4]. From a pathological point of view, coronary stenosis is primarily responsible for the extension of the inflammatory processes of intima proliferation and contraction of the fibrotic media and adventitia from the ascending aorta [5]. The formation and accelerated progression of atherosclerosis triggered by vascular inflammation may be an alternative interpretation of the coronary artery lesions [6, 7]. In our present case, IVUS revealed mild intimal hyperplasia at the ostium of the LMCA that developed negative remodelling with severe stenosis. Due to the unavailability of histological examination, the pathological mechanisms linking Takayasu arteritis and coronary artery negative remodelling are unknown and need further investigation.

IVUS can help to identify the different structures of the coronary artery wall and plaque properties of coronary lesions. However, there are only individual case reports concerning the application of IVUS in patients with Takayasu arteritis. Takeshi et al. reported unrecognized media and the expansion of fibrotic thickening from the media to the adventitia in Takayasu arteritis patients [8]. Klaus et al. reported several hypoechogenicities in the wall of coronary artery [9]. In marked contrast, our case presented negative remodelling at the ostium of the LMCA.

The physiopathology of Takayasu arteritis has not been established with certainty. However, the observation that it mostly involves cellular immunity has been generally accepted [10, 11]. T-cell-dependent immunity, chemokine- and cytokine-dependent immunity, and uncertain B-cell-dependent immunity are the main pathogeneses of arterial wall damage [11]. Thus, glucocorticoids comprise the mainstay of therapy for the induction and maintenance of inflammatory remission in patients with Takayasu arteritis, and nonbiological disease-modifying agents are usually administered in combination with glucocorticoids due to the higher relapse rates [12]. There are also few previous reports indicating whether negative remodelling persists after Takayasu arteritis treatment. However, published reports have suggested that arterial stenosis caused by Takayasu arteritis can be reversed after immunosuppressive therapy [13–15]. For coronary lesions, alleviated left main coronary ostium stenosis has been reported following corticosteroid therapy and coronary artery bypass grafting [16, 17]. Mohan et al. reported reduced stenotic lesions of the coronary ostia by steroid treatment in an 11-year-old boy with the aid of cardiac-gated computed tomography [18]. Tetsuro et al. reported attenuated coronary ostial stenosis via coronary angiography with immunosuppressive combination therapies [19]. As described in our case, an increased EEM area of the ostium of the LMCA was observed by longitudinal evaluation of IVUS at different time points after PTCA and immunosuppressive therapy.

The optimal therapeutic approach has not been defined due to the infrequency of coexistence of Takayasu arteritis with coronary artery lesions. Generally, percutaneous coronary intervention is an off-label indication that is not currently recommended for the revascularization of active Takayasu arteritis with coronary stenosis [12]. Angiography itself is an invasive procedure that may increase the likelihood of iatrogenic damage to vessels with persistent inflammation. In addition, patients with active disease appear to be more likely to necessitate surgical reconstruction for or develop restenosis in arterial lesions of Takayasu arteritis [20]. However, critical vascular ischaemia still warrants urgent intervention.

Our case showed that it is feasible to perform PTCA alone to reduce severe stenosis and postpone stent implantation for further evaluation during follow-up. Our case could provide an alternative treatment strategy for patients with Takayasu arteritis who have critical coronary ischaemia but are unsuitable or unwilling to undergo coronary artery bypass grafting.

In conclusion, we found that coronary negative remodelling might be reversible in patients with Takayasu arteritis.

## Data Availability

Data are available from Shiqiang Zhou (email: 532327195@qq.com) upon reasonable request and with permission by Xi’jing Hospital.

## Abbreviations

IVUS: Intravascular ultrasound
LMCA: Left main coronary artery
RCA: Right coronary artery
PTCA: Percutaneous transluminal coronary angioplasty
EEM: External elastic membrane
